# High grade renal trauma due to blunt injury in children: do all require intervention?

**DOI:** 10.1590/2175-8239-JBN-2018-0186

**Published:** 2019-01-10

**Authors:** Krishna Kumar Govindarajan, Mallikarjun Utagi, Bikash Kumar Naredi, Bibekanand Jindal, Kumaravel Sambandan, Deepakbharathi Subramaniam

**Affiliations:** 1 Jawaharlal Institute of Postgraduate Medical Education & Research Departments of Pediatric Surgery & Radiology Pondicherry605006 India Jawaharlal Institute of Postgraduate Medical Education & Research, Departments of Pediatric Surgery & Radiology, Dhanvantri Nagar, Pondicherry 605006, India.

**Keywords:** Wounds, Nonpenetrating, Accidents, Traffic, Kidney, Pediatrics, Treatment Outcome, Conservative Treatment

## Abstract

**Introduction:**

The aim of this study was to analyze the presentation and management of major grade renal trauma in children.

**Method:**

A retrospective study was performed including data collected from the patients who were admitted in Pediatric surgery with major grade renal injury (grade 3 and more) from January 2015 to August 2018. Demography, clinical parameters, management, duration of hospital stay and final outcome were noted.

**Results:**

Out of 13 children (9 males and 4 females), with age range 2-12 years (mean of 8 years), reported self-fall was the commonest mode of injury followed by road traffic accident. The majority (10/13, 75%) had a right renal injury. Eight children had a grade IV injury, one had a grade V injury, and four children had grade III injury. Duration of hospital stay varied from 3 to 28 (mean of 11.7) days. Three children required blood transfusion. One child required image guided aspiration twice and two required pigtail insertion for perinephric collection. All the 13 children improved without readmission or need for any other surgical intervention.

**Conclusion:**

Children with major grade renal trauma due to blunt injury can be successfully managed without surgical intervention and minimal intervention may only be needed in select situations.

## INTRODUCTION

Blunt abdominal injury in children usually involves solid organs such as liver and spleen. Kidneys are less commonly injured, estimated to be involved in around 20% of the children with blunt abdominal injury^1^. The optimal management of renal injury has been the subject of debate for a long time. Operative management for high grade kidney injury has not been shown to increase recovery and paradoxically higher rates of nephrectomy are recorded in the literature. Conservative management is the current standard of care adopted in abdominal solid visceral injuries of liver and spleen. Hence, there is a general shift towards the non-operative management of renal injury, similar to the successful outcome observed in the management of liver and spleen injuries^2^.

Most low grade injuries of the kidney are eminently responsive to observation without intervention. But whether the same conservative mode can be extrapolated to the high grade injuries, which may be associated with a critically ill status, requires evaluation. This study was planned to analyze outcomes of high grade blunt renal injury in children.

## METHODOLOGY

A retrospective chart review was conducted in our department (a tertiary care University Teaching Hospital) to identify the data of the children admitted with high grade blunt renal trauma from January 2015 to August 2018. Relevant findings including mode of injury, clinical parameters, imaging findings, laboratory parameters, intervention, and outcome were noted (Table 1). Grading of renal injury was reported as per the American Association for the Surgery of Trauma (AAST) injury scoring scale^3^. The hospital stay included observation with close monitoring of vital signs and relevant medications such as analgesics and intravenous antibiotics. After discharge, the children were followed up at 2-4 monthly intervals with imaging and outpatient visits.

**Table 1:** Data of children with high grade renal trauma

	Age (in years)	Gender	Side	Mode of injury	Grade of injury	Intervention	Associated organ injury	Duration of hospital stay (in days)	Outcome
1	9	F	Right	self-fall	4	nil	nil	9	well
2	9	F	Right	self-fall	4	Aspiration	nil	16	well
3	5	M	Right	self-fall	4	Pigtail drain	nil	28	well
4	11	M	Left	RTA	4	nil	Spleen	3	well
5	11	M	Left	RTA	4	nil	Spleen	14	well
6	7	M	Right	RTA	4	nil	Liver	7	well
7	12	F	Right	self-fall	3	nil	nil	3	well
8	9	M	Right	self-fall	4	Pigtail drain	nil	14	well
9	12	M	Left	RTA	3	nil	nil	8	well
10	10	M	Right	self-fall	5	nil	Spleen	25	well
11	11	M	Right	self-fall	4	nil	nil	14	well
12	2	F	Right	RTA	3	nil	nil	7	well
13	3	M	Right	RTA	3	nil	nil	4	well

RTA - Road traffic accident

## RESULTS

**Figure 1:**
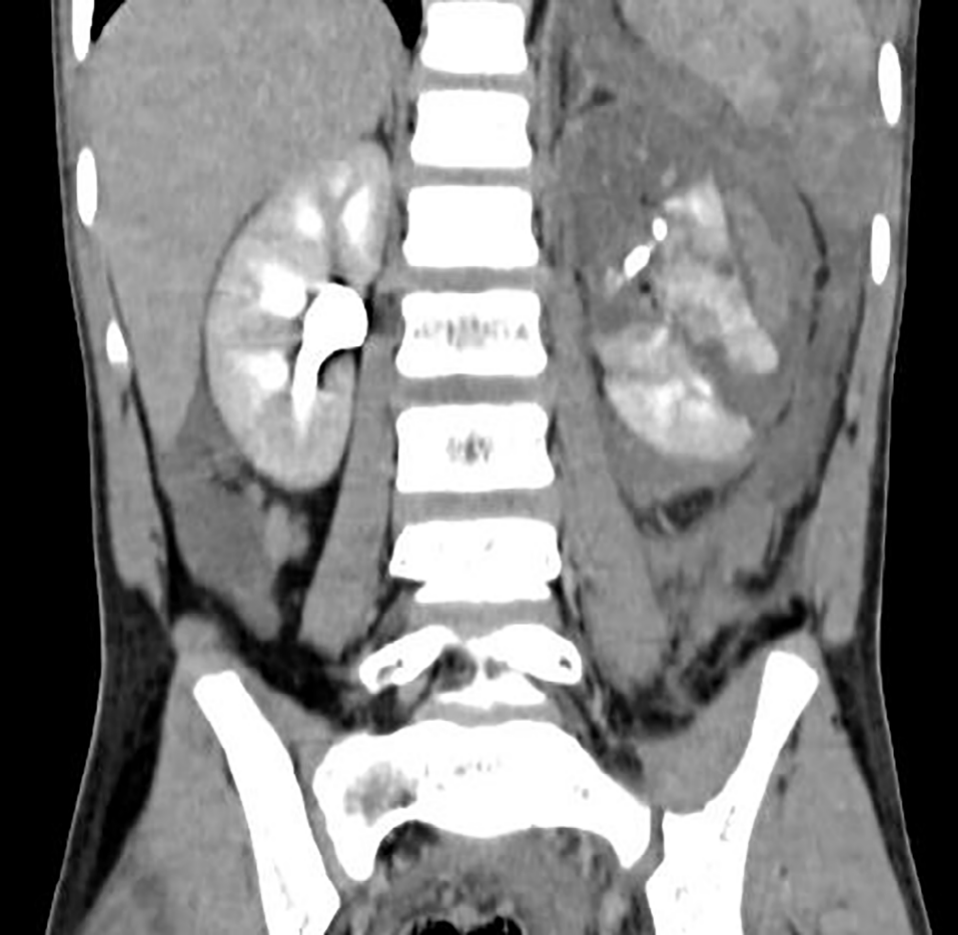
Combined splenic injury and Grade-4 left renal injury.

**Figure 2:**
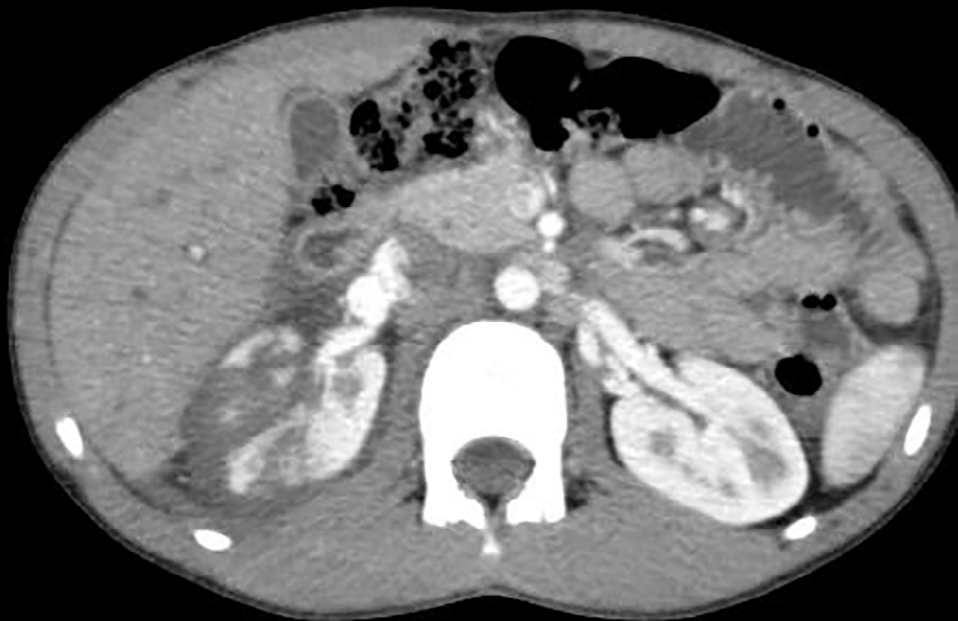
Isolated right renal injury (Grade-3).

**Figure 3:**
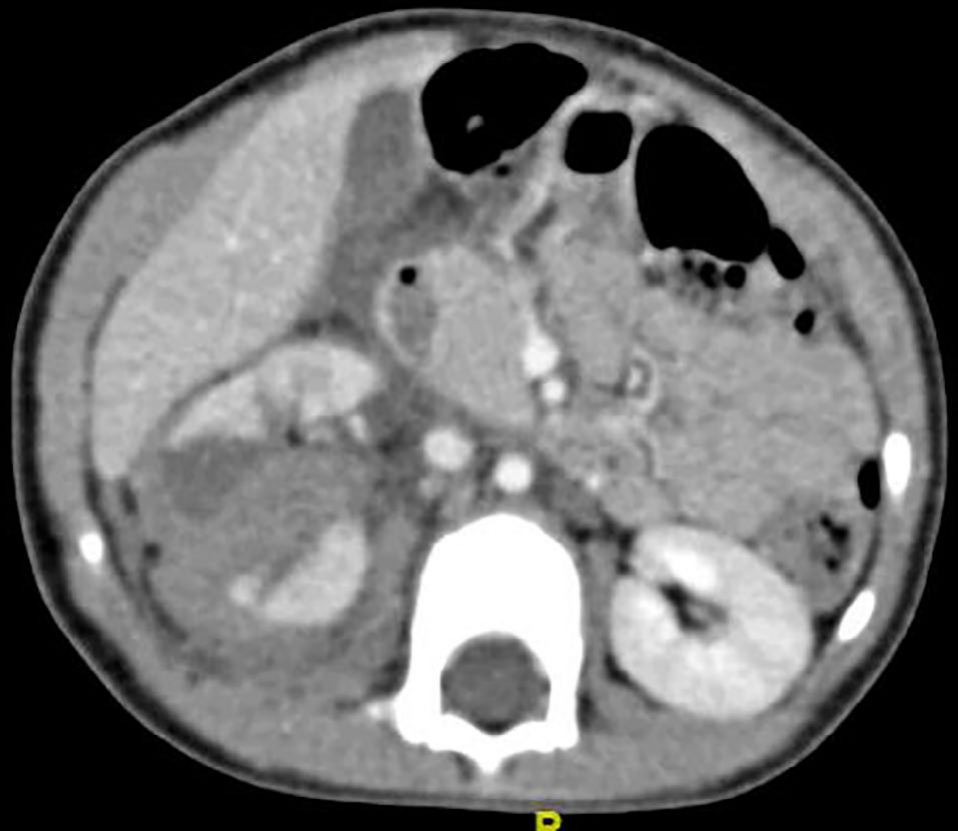
Right renal injury (Grade 5 - shattered renal parenchyma with contained hematoma)

A total of 13 children were admitted with high grade renal injury (grade 3 and above) during the study period, of whom 9 were boys and 4 were girls, age ranging from 2 to 12 years. The most common mode of injury was self-fall noted in 7 children and road traffic accident in 6 children. Associated organ injuries were seen in 4 of 12 children, of whom 3 had injury to spleen (Figure 1) and one had a liver injury. Ten children had right sided renal injury and 3 had left renal injury. All of them presented with a history of trauma with abdominal pain followed by vomiting and hematuria. In our study, the majority were grade 4 injury in 8 cases followed by 4 children with grade 3 injury (Figure 2) and one with grade 5 injury (Figure 3). Mean hospital stay was 11.7 days (range 3 to 28 days), 6 children had persistent febrile spikes during the hospital stay, of whom 3 required interventions and the rest settled spontaneously. Blood transfusion was carried out in 3 children due to falling hemoglobin, which stabilized after the initial transfusion. Overall, only 3 of 13 children with high grade renal injury needed interventions namely sonography-guided aspiration in one and pig-tail drain insertion in two children. The resultant aspirate was urine in all the 3 children. All children recovered well without the need for further interventions or procedures. The children were seen in the out patients clinic at regular intervals of 3-4 months for a period of at least 1 year after trauma, after which the follow-ups continued annually.

## DISCUSSION

Children are at increased risk for renal trauma owing to the unique anatomic differences compared to adults. In children, certain anatomic characteristics make them more exposed and vulnerable to injury, such larger kidneys relative to the size of the child’s body, kidneys positioned lower in the abdomen, and less protected because of less peri-renal fat, weaker abdominal wall musculature, and a less calcified thoracic rib cage. Furthermore, the renal capsule and Gerota’s fascia are less developed than in adults, creating a greater potential for parenchymal laceration, non-confined bleeding, and urinary extravasation. Mechanisms of blunt renal injury include pedestrian/motor vehicle crashes (60%), falls (22.5%), sports injuries (10%), assault (3.5%), and other causes (4%)^4^.

In contrast to adults, hematuria in children is a very unreliable sign in determining the need to screen for renal injures. In some studies, there is no evidence of gross or microscopic hematuria in up to 70% of children sustaining grade 2 or higher renal injury. As the clinical features may not be a reliable indication of the severity of the visceral injury, a through imaging protocol of sonography and contrast enhanced computed tomography scan would be in order, to avoid delay in the diagnosis of renal injury^5^.

The majority of isolated renal injuries can be classified as relatively minor injuries. Mortality is rare from isolated renal trauma and is more often attributed to the combined effects of major multisystem trauma. The ultimate goal of management of renal injury is to maximize functional renal parenchyma and minimize patient morbidity. Advances in radiographic staging of the renal injury severity assessment help to accurately prognosticate and plan management strategies^1^^,^^2^.

Renal injuries are graded on a scale from I to V ranging from minor (grade I) to most complex (grade V) as per the AAST. Traditionally, grade I to grade III injuries have been successfully managed non-operatively, while grade V injuries are submitted to surgical exploration and repair. For patients with grade IV renal injury, the role and timing of surgical, endourologic, and radiographic intervention is less established and remains controversial^6^. However, data from recent studies suggest conservative management for even high-grade pediatric blunt renal trauma. The successful management rates range from 40 to 84%^7^. When blunt trauma is accompanied by significant urinary extravasation, interventions such as percutaneous drainage, guided aspiration and internal stenting, may be required to tackle the complications due to extravasation of urine. On the contrary, most of the surgical explorations as per contemporary management for high grade renal trauma resulted in nephrectomy. When possible, non-operative management in similar cases would result in increased rates of salvage and subsequent renal preservation^8^.

In the present study, the majority were grade-4 renal injuries, with self-fall as the common mode of injury. All children were hemodynamically stable. Excluding three children who underwent intervention in the form of pigtail drain insertion and percutaneous aspiration for progressively increasing perinephric urinoma and high spiking fever, none required any major surgical intervention. Also, all of them were doing well on follow up after trauma.

The long term consequences have not been addressed in our study owing to the on-going follow up of these children. It has been proposed that onset of hypertension and altered renal functions are consequences to previous high grade renal trauma^9^^,^^10^.

Although the number of patients in this study was small, the study assumed significance owing to the uncommon incidence of major grade renal trauma in children.

## CONCLUSION

Advances in imaging, improvements in hemodynamic monitoring, validated renal injury scoring systems, and accurate details about the mechanisms of injury allow successful non-operative management strategies for renal preservation. Non-operative treatment can be safely recommended in children with high grade renal trauma, provided there is no continuing hemorrhage and hemodynamic stability is achieved. Selective intervention in the form of percutaneous drainage may be required in a minority of patients, to help in hastening the complete resolution of urine leakage when blunt trauma is accompanied by significant urinary extravasation.
